# Amniotic Stem Cells: Potential in Regenerative Medicine

**DOI:** 10.1155/2012/530674

**Published:** 2012-12-02

**Authors:** Mahmud Bani-Yaghoub, Patricia Wilson, Markus Hengstschläger, Toshio Nikaido, Duanqing Pei

**Affiliations:** ^1^Neurogenesis and Brain Repair, National Research Council Canada-Institute for Biological Sciences, Bldg. M-54, Ottawa, ON, Canada K1A 0R6; ^2^Department of Cellular and Molecular Medicine, Faculty of Medicine, University of Ottawa, Ottawa, ON, Canada K1H 8M5; ^3^Wake Forest Institute for Regenerative Medicine, Wake Forest Baptist Health, Medical Center Boulevard, Winston-Salem, NC 27157, USA; ^4^Institute of Medical Genetics, Medical University of Vienna, Währinger Straße 10, 1090 Vienna, Austria; ^5^Department of Regenerative Medicine, University of Toyama Graduate School of Medicine and Pharmaceutical Sciences, 2630 Sugitani, Toyama 930-0194, Japan; ^6^Key Laboratory of Regenerative Biology, South China Institute for Stem Cell Biology and Regenerative Medicine, Chinese Academy of Sciences, 190 Kai Yuan Avenue, Science Park, Guangzhou 510530, China

This special issue of *Stem Cells International* focuses on the potential applications of amniotic cells in regenerative therapies to repair tissues and organs that have been damaged by trauma, disease, or aging. Amniotic cell populations have historically included cells in amniotic fluid that have been sloughed from external and internal surfaces of the developing fetus and potentially from the amnion, the innermost extraembryonic membrane of the amniotic sac. Amniotic fluid is recovered by amniocentesis, a technique that was initially used to assess fetal health in cases of Rh blood group incompatibility [1] and to determine the sex of the fetus by the presence or absence of the Barr body in amniotic cells [2, 3]. These pioneering studies [4] provided accessible methods for *ex vivo* culture of amniotic cells as a source of normal, rather than transformed cancer cells for biomedical research. Fauza and colleagues [5] were among the first to explore use of amniotic cells for tissue engineering and repair of congenital defects *in utero*. Ovine amniotic fluid and later human amniotic fluid proved to contain populations of rapidly proliferating mesenchymal, also known as stromal, cells that have since dominated research efforts. Similar to multipotential stromal cells (MSCs) derived from bone marrow [6, 7] and adipose tissue [8, 9], amniotic stromal cells differentiate into connective tissue lineages and show beneficial trophic support and immunomodulatory activities that promote self-repair [10–13]. Trophic support and immunomodulatory activities are not unique to stromal cells; several active clinical trials are testing for the safety and efficacy of amniotic membrane derived epithelial cells that show similar properties (http://www.ClinicalTrials.gov/).

Unique features of amniotic cells present opportunities for regenerative medicine and biomedical research that are not shared by other cell sources, including pluripotent stem cells as defined by the International Society for Stem Cell Research (http://www.isscr.org/). A unifying feature of amniotic and placental cell sources is *ex vivo* culture from some of the earliest stages of fetal development, ranging from 15 weeks to 17 weeks of gestation with amniocentesis sampling to near birth at 9 months with delivery of full term placenta. Another unique feature reflects signaling cascades during gestation; amniotic and placental cells are exposed to paracrine signaling among the mother, the fetus, and the placenta. These transient conditions may confer distinctive properties to amniotic cells that are not shared by other cell sources. The consensus of the diverse papers offered here is that continued research and development may prove amniotic cells to be superior cell sources for selected applications. 

Further progress would be facilitated with identification of useful biomarkers for amniotic cells. In a review article by M. G. Roubelakis et al. in Greece “Amniotic fluid and amniotic membrane stem cells: marker discovery,” readers are provided with the most recent approaches used to investigate stem cells in amniotic membrane and fluid. The authors have assembled a comprehensive list of markers employed to analyze these cells with the techniques focused on transcriptomics, proteomics, and metabolomics. The authors further emphasize the importance of developing efficient methods to isolate homogeneous stem cell populations prior to their use for cell-based therapies. 

Biomarker identification is furthered by a research article by M. P. Dobreva et al. in Belgium and the Netherlands “Periostin as a biomarker of the amniotic membrane” examining the target genes of bone morphogenetic proteins (BMP) as amniotic membrane markers. Comparative gene expression analyses combined with *in situ hybridization* assays presented in this paper has led to the identification of periostin as a reliable marker expressed in the amnion throughout gestation. Furthermore, the authors have established a transcriptional signature for extraembryonic tissues, using a carefully selected panel of genes, encouraging the development of other biomarkers in this area. 

While the first two papers in this special issue have focused on biomarkers, a review article by R. J. Hodges et al. in Australia presents “Amnion epithelial cells as a candidate therapy for acute and chronic lung injury”. The authors discuss the plasticity and immunomodulation properties of amnion epithelial cells in repair lung injury. In addition, they address the therapeutic potential of these cells with references to purity of cell population and the transcription factors they express. Key features of amnionepithelial cells and their role as exogenous stem cells compared to resident endogenous lung stem cells have been presented.The authors provide a list of factors that are potentially involved in the repair mechanisms elicited by amnion. 

In a paper focused on mesenchymal stem cells, C. M. Raynaud et al. in Qatar and USA. present a “Comprehensive characterization of these cells from human placenta and fetal membrane and their response to osteoactivin stimulation.” The authors have employed transcriptomics, immunocytochemistry, cytokine array analysis, and other complementary methods to evaluate the similarities and differences between placenta- and membrane-derived mesenchymal stem cells before and after differentiation. Using established protocols to isolate these cells from both sources, the authors show that placenta- and membrane-derived mesenchymal stem cells respond differently to osteoactivin during osteogenic differentiation. 

A concise review of “Amniotic fluid stem cells: future perspectives.” By M. Rosner et al. in Austria discusses the progress made by this group as well as others, since their first report on connection between amniotic fluid cells and stem cell research. The comparison among AFS cells, ES cells, adult stem cells, and iPS cells has been followed by an emphasis on the importance of monoclonal genomically stable human AFS cell lines and their capacity to form embryoid bodies. As a novel approach, this paper has regarded AFS cells as a source for cell-based therapies for kidney disorders such as acute and chronic renal failures and acute tubular necrosis. While promising, the authors have emphasized that standardized protocols are required to isolate monoclonal populations of AFS cells. 

The challenge inherent to population complexity in amniocentesis samples as well as the potential effects of paracrine signaling prompted P. G. Wilson et al. in the USA, “Clonal populations of amniotic cells by dilution and direct plating: evidence of hidden diversity,” to first establish an efficient method to generate amniotic cell cultures, avoiding the need for refrigeration and centrifugation of samples. Application of this method allowed isolation of a large number of clonal lines from independent amniocentesis samples. As proof of concept for clonal identity, clonal populations of stromal and epithelial cells were compared with each other and with control cell populations. The results of flow cytometry, immunocytochemistry, differentiation tests, and transcript analysis revealed clear differences not only among stromal cell clones and epithelial cell clones from the same donor, but also between mixed cell populations from different donors. 

Functional communication between the grafted and host cells plays a pivotal role in delivering therapeutic agents to the damaged tissues. In a research paper by A. Jezierski and colleagues in Canada “Human amniotic fluid cells form functional gap junctions with cortical cells,” the authors show that amniotic fluid cells express high levels of connexin 43, a gap junction protein known for its role in brain development and function. Furthermore, AF cells establish intercellular communication with astrocytes, suggesting a novel role for AF cells to deliver therapeutic factors, including specific microRNAs and small molecules, to the damaged tissues. In particular, this approach may facilitate neuroprotection in the early stages of graft-host interaction during which intercellular coupling via gap junctions precedes the integration of transplanted cells into the neuronal circuit.

A key feature of this special issue of *Stem Cells International* is to provide an overview of the recent progress in the field of amniotic stem cells by independent research groups from different continents. In an article by K. Rennie et al., authors from Canada, Austria, China, and Japan discuss applications of amniotic membrane and fluid in stem cell biology and regenerative medicine. While amniotic membrane is widely used as a natural scaffold for clinical applications, using standardized protocols to establish homogeneous clones from amniotic membrane or fluid cells has been described as a critical step towards development of desired phenotypes for cell transplantation. The paper has also recapitulated the use of amniotic fluid cells in several disease models, hoping that more progress will be made towards effective regenerative therapies in the near future.

The editors hope that the original and review articles integrated in this special issue provide more insights into the advancement and challenges associated with the use of amniotic cells in regenerative medicine.

*Mahmud Bani-Yaghoub* *Mahmud Bani-Yaghoub* *Patricia Wilson* *Patricia Wilson* *Markus Hengstschl$\ddot{\text{a}}$ger* *Markus Hengstschl$\ddot{\text{a}}$ger* *Toshio Nikaido* *Toshio Nikaido* *Duanqing Pei* *Duanqing Pei*
